# Development of a European competency framework for health and other professionals to support behaviour change in persons self-managing chronic disease

**DOI:** 10.1186/s12909-021-02720-w

**Published:** 2021-05-20

**Authors:** Mara Pereira Guerreiro, Judith Strawbridge, Afonso Miguel Cavaco, Isa Brito Félix, Marta Moreira Marques, Cathal Cadogan

**Affiliations:** 1 Nursing Research, Innovation and Development Centre of Lisbon (CIDNUR), Nursing School of Lisbon, Lisbon, Portugal; 2 Centro de Investigação Interdisciplinar Egas Moniz (CiiEM), Instituto Universitário Egas Moniz, Monte de Caparica, Portugal; 3 Royal College of Surgeons in Ireland, School of Pharmacy and Biomolecular Sciences, Dublin, Ireland; 4 Faculty of Pharmacy, University of Lisbon, Lisbon, Portugal; 5 ADAPT SFI Research Centre & Trinity Centre for Practice and Healthcare Innovation, Trinity College Dublin, Dublin, Ireland; 6 Comprehensive Health Research Centre (CHRC), NOVA Medical School, Lisbon, Portugal; 7 Trinity College Dublin, School of Pharmacy and Pharmaceutical Sciences, Dublin, Ireland

**Keywords:** Interprofessional, Competency framework, Behaviour change, Behaviour change techniques, Chronic disease, Education

## Abstract

**Background:**

Healthcare and other professionals are expected to support behaviour change in people living with chronic disease. However, effective behaviour change interventions are largely absent in routine encounters. The Train4Health project, a European strategic partnership for higher education, sought to address this problem. The primary aim of this study, which is part of an early work package, was to develop an interprofessional competency framework for health and other professions to support behaviour change for the self-management of chronic disease at a European level. A secondary aim was to derive a set of behaviour change techniques (BCTs) from an established taxonomy to link with framework competencies.

**Methods:**

The study comprised two interlinked parts. Part 1 involved a two-round e-Delphi study with an interprofessional panel of 48 experts across 12 European countries to develop the behaviour change competency framework. Preparatory work included drafting a list of competency statements based on seven existing frameworks.

Part 2 involved an expert panel of six behavioural psychologists deriving a set of BCTs to link with framework competencies. Their feedback was based on preparatory work, which focused on seven high priority chronic diseases for self-management, identified through European projects on self-management and identifying five relevant target behaviours from key clinical guidelines. A literature search yielded 29 effective BCTs for the target behaviours in the selected chronic diseases.

**Results:**

Twenty-seven competency statements, were presented in Round 1 to the Delphi panel. Consensus was achieved for all statements. Based on comments, two statements were removed, one was added, and 14 were modified. All 15 statements subjected to Round 2 were consensus-approved, yielding a total of 12 foundational competencies for behaviour change in self-management of chronic disease and 14 behaviour change competencies. Four behaviour change competencies related to BCTs. Behavioural psychologists’ feedback led to a core set of 21 BCTs deemed applicable to the five target behaviours across the seven chronic diseases.

**Conclusions:**

A behaviour change competency framework comprising 26 statements for European health and other professionals to support self-management of chronic disease was developed, linked with a core set of 21 BCTs from an established taxonomy.

**Supplementary Information:**

The online version contains supplementary material available at 10.1186/s12909-021-02720-w.

## Background

Chronic diseases, also known as non-communicable diseases, are a global epidemic, responsible for 40.5 million deaths in 2016, corresponding to 71% of deaths worldwide [1]. Cardiovascular diseases, cancers, diabetes and chronic lung diseases present the highest prevalence, which is expected to increase in the coming years [2]. Addressing chronic diseases is one of the United Nations key sustainable development goals [3]. Changing and sustaining desirable lifestyle behaviours are critical to achieving this goal, both from a prevention and treatment standpoint.

Self-management is defined as tasks performed by an individual to minimize the impact of one’s disease, with or without the support of health professionals. Tasks can holistically be categorized under medical management (e.g. taking medication, adhering to a diet, engaging in physical activity), role management (e.g. redefining life roles in light of a chronic disease) and emotional management (e.g. dealing with anger and frustration) and are related to a set of skills [4]. This definition captures the idea that self-management encompasses a variety of health behaviours in which individuals should engage.

Healthcare and other professionals are expected to support behaviour change for the self-management of chronic disease, including, for instance, interventions to improve diet or increase physical activity. However, effective interventions targeting a range of health behaviours are still not the norm in routine encounters. For example, a qualitative study with healthcare professionals working in the UK’s National Health Service revealed that professionals perceived a lack of confidence in their own skill set and ability to implement behaviour change interventions [5]. Another study, conducted in Dutch primary care, concluded that nurses tended to prioritise the optimisation of medical treatment and seldom focused on behaviour change [6]. Furthermore, competent behaviour change counselling was regarded as uncommon in clinical practice in Canada [7]. Overall, this evidence suggests the existence of a global workforce problem in respect of perceived knowledge and skills relating to the implementation of behaviour change interventions.

Behaviour change techniques (BCTs; e.g. setting goals, self-monitoring of behaviour, social support) represent an attempt to unpack the black box of behaviour change interventions. A BCT is “an observable, replicable, and irreducible component of an intervention designed to alter or redirect causal processes that regulate behavior” [8]. Michie et al. previously attempted to derive behavioural competencies for professionals supporting smoking cessation based on evidence of efficacy of BCTs and guidance documents [9]. More recently, seminal British guidance on individual-level health behaviour change interventions recommended detailing the BCTs by using a taxonomy, so that interventions can be replicated and include techniques shown to be effective at changing behaviour [10]. This guideline from the National Institute for Health and Care Excellence (NICE) has recommended that behaviour change practitioners should recognise BCTs in the intervention they are delivering and have the relevant skills to deliver them [10]. The BCT Taxonomy (version 1 - BCTTv.1) has gained international acceptance as a tool for specifying the content of behaviour change interventions [8, 11]. Notably, a recent scoping review found that BCTs remain underused in self-management interventions [12]. One reason that may explain this shortcoming is the poor permeation of behavioural science, and BCTs in particular, into the education and training of health and other professionals.

Train4Health (https://www.train4health.eu) is a strategic partnership involving seven European Institutions across five countries, which seeks to improve behaviour change support competencies for the self-management in chronic disease. The Train4Health consortium comprises Institutions involved in the education of nursing students, pharmacy students, sports sciences students, an IT partner and the European Students’ Union. Hallmarks of the Train4Health project (2019 -) include drawing on behavioural science and co-production with users of educational products. The project envisages a continuum in behaviour change support education, in which an interprofessional competency framework, relevant for those currently practising, guides the development of a learning outcomes-based curriculum and an educational package for future professionals (today’s undergraduate students). The educational package, comprising case studies, a massive open on-line course and a simulation software package, that will align with relevant European Union policy on digital transformation in education and training.

Pursuing the Train4Health aim required an interprofessional competency framework agreed across disciplines and European countries, focused on self-management in chronic disease and linked to a set of BCTs from a standardised taxonomy [8, 11]. Existing health behaviour change competency frameworks [7, 13–18] did not respond to these cumulative requirements. For example, none were linked to BCTs from current taxonomies or, when including BCTs, made explicit the process underlying their selection. Most importantly, none of these competency frameworks resulted from a transnational consensus process.

The primary aim of this study was to develop an interprofessional competency framework for health and other professions to support behaviour change for the self-management of chronic disease at a European level. A secondary aim was to derive a set of standardized BCTs to link with framework competencies that directly support behaviour change.

## Methods

In this section, the method used to address the primary and secondary study aims are described in turn. In essence, a Delphi method was used to consensualise the behaviour change competency framework, based on a draft list of competencies compiled from existing frameworks (Part 1). Deriving a set of BCTs to be linked with framework competencies was achieved through a combination of a literature search with feedback from an expert panel of behavioural psychologists (Part 2).

All methods were carried out in accordance with relevant guidelines and regulations.

### Part 1: development of the behaviour change competency framework

The Delphi technique is a widely used method for achieving consensus of opinion from experts within a particular field [19]. It allows stakeholders’ views and experiences to be captured as part of a consensus-building exercise [20]. The study methodology outlined below is adapted from previous Delphi studies [21, 22]. Ethical approval was granted by the RCSI Research Ethics Committee (REC201911014).

#### Preparatory work

##### Compiling a draft list of competencies for inclusion in the Delphi study

Members of the research team reviewed existing health behaviour change competency frameworks to inform the initial draft list of competencies [7, 13–18], herein designated as “reference documents”. Competencies that were included in, or derivable from, these reference documents were identified and extracted. Each competency was drafted as a statement of the activity that the healthcare professional is required to undertake (e.g. ‘knowledge of’, ‘ability to’). The competencies were initially categorised based on whether they were primarily knowledge or skills focused.

The drafted competencies were compared across the reference documents to create a single merged long-list of 47 competencies (Additional file 1). Each competency framework that included, or from which each competency was derived, was recorded in a tabular format, to assist in determining where there was some level of agreement in the reference documents. The long-list was prepared by one member of the research team and reviewed by another member for accuracy and completeness. The research team subsequently reviewed and refined this long-list of competencies, retaining 25 included in, or derived from, three or more competency frameworks. The remainder were included or excluded based on discussion among the research team. Statements that were not identified as part of the scoping exercise, but which were deemed to be of importance, such as competencies on BCTs, were added where appropriate. A refined list of 27 competencies was then circulated to the wider Train4Health consortium for review. To provide greater clarity, the competencies were divided into two categories (1) competencies that directly support behaviour change in the self-management of chronic disease, and (2) foundational competencies required for effective delivery of behaviour change support. The final refined list, comprising 27 competency statements, was recirculated for approval by the research team, and inclusion in Round 1 of the Delphi study.

#### Delphi consensus on competencies

##### Specification of the target population

The competency framework was developed for health and other professions, using the pharmacy, nursing and exercise physiologists’ groups as a starting point. These disciplines are representative of the Train4Health consortium and can contribute to self-management behaviours in chronic disease (e.g. medication adherence, smoking cessation, physical activity, weight loss), both individually and collectively.

##### Delphi panel selection

For the purpose of this study an individual was considered an expert if the following criteria was fulfilled:

- Being involved in either behaviour change support education in chronic disease or in delivering behaviour change support in practice and,
- Professional credentials (e.g. track-record in the field evidenced by publications or professional experience) and/or status (e.g. job title) within each group.

As there is no universally agreed sample size for Delphi studies [23], the sampling strategy followed a maximum variability approach and sought to obtain a range of perspectives from academic educators and healthcare professionals with backgrounds in relevant disciplines (e.g. pharmacy, nursing, sports sciences). Eighty individuals across European countries complying with the aforementioned definition of “expert” were suggested by members of the Train4Health consortium and invited to take part in the study. Eight additional individuals were invited during Round 1 based on the recommendation of those initially invited.

##### Data collection and analysis

The Delphi study comprised two rounds of online questionnaires. The initial questionnaire was piloted using a convenience sample of academics from the Train4Health consortium, to check the questionnaire’s face validity and the usability of the online survey software tool SurveyGizmo® (Additional file 2). These responses were not included in the final analysis.

Round 1 of the Delphi study took place between June and July 2020 and Round 2 took place in August 2020. During each round, panellists received an email with a link to the online questionnaire together with instructions on completing it. Panellists also received a glossary of key terms used throughout the questionnaire (Additional file 3). Up to two email reminders per participant in each round were employed to maximise the response rate.

In line with previous Delphi studies [21, 22], panellists used a 5-point Likert scale to rate their level of agreement with each statement (1 = strongly disagree, 5 = strongly agree). For each statement, the median response value and interquartile range was calculated. Statements were then rejected or included in Round 2 of the Delphi study using a priori consensus rules:

- A lower quartile ≥4 indicated consensus amongst panellists and the statement was accepted (consensus “in”).
- An upper quartile ≤2 indicated disagreement and the statement was rejected (consensus “out”).
- If the interquartile range included 3, this indicated a lack of agreement amongst panellists and a need for further review of the particular statement (no consensus). In the event of such cases, the statements were to be reviewed by the research team and either revised and included in the next round of the Delphi study or rejected based on the panellists’ additional comments.

In Round 1, participants had the opportunity to add free-text comments to each of the statements and to suggest additional statements for inclusion in the questionnaire. In Round 2, participants were provided with a summary of Round 1 scores showing summary group-level statistics for each statement’s rating. The same analysis and application of consensus rules was undertaken as per Round 1.

### Part 2: deriving a set of BCTs to be linked with framework competencies

#### Preparatory work

Applying BCTs in the context of chronic disease requires addressing specific behaviours in persons living with chronic conditions, and identifying which BCTs are associated with greater effectiveness in this context. As articulated by NICE guidance, “being trained to deliver one behaviour change intervention does not necessarily mean that a practitioner is then competent to deliver other behaviour change interventions” [10]. These considerations set the rationale for the steps detailed below: narrowing chronic diseases (firstly) to those recognised as high priority for self-management, detailing target behaviours and identifying BCTs for which evidence of effectiveness existed in relation to specific behaviours in these populations.

##### Selecting high priority chronic diseases and relevant target behaviours

Seven high priority chronic diseases were identified based on two European Union (EU) funded projects addressing self-management: COMPAR-EU [24] and PRO-STEP [25]:

- Type 2 diabetes, chronic obstructive pulmonary disease (COPD), obesity, heart failure [24];
- Asthma, hypertension and ischemic heart disease [25].

Then, key international clinical guidelines [26–32] were searched to identify target behaviours relevant for the self-management of each chronic disease. The authors used their knowledge and experience of disease management to select up-to-date European or internationally recognised clinical guidelines, such as those issued by the European Society of Cardiology [28, 29, 32], the Global Initiative for Chronic Obstructive Lung Disease (GOLD) [27], the Global Initiative for Asthma [31] and the American Diabetes Association [26]. Table 1 depicts key target behaviours for the self-management of each of these high priority chronic diseases.

**Table 1:** Target behaviours for the self-management of high priority chronic diseases

Behaviour	Type 2 diabetes [26]	COPD [27]	Hypertension [28]	Heart failure [29]	Obesity [30]	Asthma [31]	Ischemic heart disease [32]
Diet (including alcohol intake)	**●**	**●**	**●**	**●**	**●**	**●**	**●**
Physical activity	**●**	**●**	**●**	**●**	**●**		**●**
Medication adherence	**●**	**●**	**●**	**●**	**●**	**●**	**●**
Smoking cessation	**●**	**●**	**●**	**●**		**●**	**●**
Symptom monitoring and management	**●**	**●**		**●**		**●**	**●**

##### Literature search on effective BCTs in high priority chronic diseases

A literature search was undertaken to identify evidence of effective BCTs to address key target behaviours in high priority chronic diseases. Due to the timeframe and available resources, only systematic reviews were considered. Inclusion criteria comprised systematic reviews of self-management intervention trials, in which BCTs were detailed, in any of the seven high priority chronic diseases. Another eligibility criterion was the use of the BCTTv1 to code BCTs [8, 11]. Studies reporting interventions targeting healthcare professionals were excluded, as well as reviews reporting clusters instead of individual BCTs.

The first search was piloted and run in PubMed, without year or language restrictions, using relevant keywords (behaviour change technique, type 2 diabetes, chronic obstructive pulmonary disease, obesity, heart failure, asthma, hypertension, ischemic heart disease) with the aid of Boolean operators and, to account for variations, the wildcard asterisk (*). The systematic review filter was employed. The search was subsequently adapted to the Cochrane Database of Systematic Reviews and the Database of Abstracts of Reviews of Effects (DARE), restricted to systematic reviews published after 2013, which was the year of publication of the Behaviour Change Techniques Taxonomy v.1 [8].

Backward and forward citation searching were conducted to identify additional records potentially eligible, by manually searching the reference list of all the reviews included and checking studies citing these reviews in Google Scholar, respectively.

Both study selection and data extraction into summary tables were performed by a single reviewer.

Five systematic reviews were identified through PubMed, targeting type 2 diabetes (*n* = 2) [33, 34], obesity (*n* = 1) [35], cardiovascular disease (*n* = 1) [36] and cardiometabolic conditions (*n* = 1) [37]. The target behaviours included in the reviews were diet, physical activity and medication adherence. The reviews covered a total of 155 studies, of which 152 were randomized controlled trials, including 68,315 patients. Reasons for excluding reviews based on full text screening were: BCTs coded with different taxonomies [38–40], no evidence of BCT effectiveness [41–43], inability to distinguish effective BCTs due to cluster analysis [44] and inability to extract data for the target diseases previously considered [45].

As depicted in Table 2, a total of 29 BCTs with evidence of effectiveness were identified for three of the five target behaviours. No evidence of effectiveness was uncovered for BCTs addressing smoking cessation and symptom monitoring and management in persons living with the high priority chronic diseases. There was a predominance of the clusters “Goals and planning” and “Feedback and monitoring” (six BCTs each). A breakdown by target behaviour showed 21 effective BCTs in diet interventions, 27 in physical activity and one in medication adherence. For the first two behaviours, there were a number of common BCTs across each of the diseases. For example, “1.2 Problem solving” was effective in promoting physical activity in type 2 diabetes, obesity and cardiovascular disease.

**Table 2:** BCTs with evidence of effectiveness for individual target behaviours in persons with high priority chronic diseases^1^ [11]

Cluster	BCT	Diet	Physical activity	Medication adherence
1.Goal and planning	1.1 Goal setting (behaviour)	**●**	**●**	
	1.2 Problem solving	**●**	**●**	
	1.3 Goal setting (outcome)	**●**	**●**	
	1.4 Action planning	**●**	**●**	
	1.5 Review behaviour goal(s)	**●**	**●**	
	1.7 Review outcome goal(s)	**●**	**●**	
2. Feedback and monitoring	2.2 Feedback on behaviour	**●**	**●**	
	2.3 Self-monitoring of behaviour	**●**	**●**	
	2.4 Self-monitoring of outcome(s) of behaviour	**●**	**●**	
	2.5 Monitoring outcome(s) of behaviour by others without feedback	**●**	**●**	
	2.6 Biofeedback		**●**	
	2.7 Feedback on outcome of behaviour	**●**	**●**	
3. Social support	3.1 Social support (unspecified)	**●**	**●**	
	3.2 Social support (practical)		**●**	
	3.3 Social support (emotional)		**●**	
4. Shaping knowledge	4.1 Instruction on how to perform a behaviour	**●**	**●**	
5. Natural consequences	5.1 Information about health consequences			**●**
6. Comparison of behaviour	6.1 Demonstration of the behaviour	**●**	**●**	
	6.2 Social comparison	**●**		
7. Associations	7.1 Prompts/cues		**●**	
8. Repetition and substitution	8.1 Behavioural practice/rehearsal	**●**	**●**	
	8.7 Graded tasks	**●**	**●**	
9. Comparison of outcomes	9.1 Credible source	**●**	**●**	
	9.2 Pros and cons	**●**	**●**	
10. Reward and threat	10.4 Social reward		**●**	
11. Regulation	11.1 Pharmacological support		**●**	
	11.2 Reduce negative emotions		**●**	
12. Antecedents	12.3 Avoidance/reducing exposure to cues for the behaviour	**●**	**●**	
	12.5 Adding objects to the environment	**●**	**●**	

^1^ BCT numbering refers to the numbering in BCTTv1

#### Expert feedback on BCTs

An expert panel of six behavioural psychologists from five countries (Canada, UK, Ireland, Finland, Portugal) was convened, all of whom were affiliated with academic and/or research institutions. The purpose of convening this panel was overcoming uncertainties and evidence gaps emerging from the previous phase. Experts were identified by the research team through published work and snowballing. Feedback was collected through a structured form. One aspect covered was generalising the evidence on effective BCTs from the conditions considered by the systematic reviews to the set of seven high priority chronic diseases considered in the project. BCTs were regarded as generalisable to this wider set of conditions if at least four experts agreed. Experts were also asked to suggest additional BCTs for the range of target behaviours, as absence of evidence on effectiveness does not necessarily equate to lack of effectiveness. Suggested BCTs were considered if at least two experts agreed.

Experts’ scoring and comments were then discussed within the research team, who included a behavioural psychologist, to reach a decision on the final list of BCTs to be linked with the framework competencies.

## Results

**Fig. 1:**
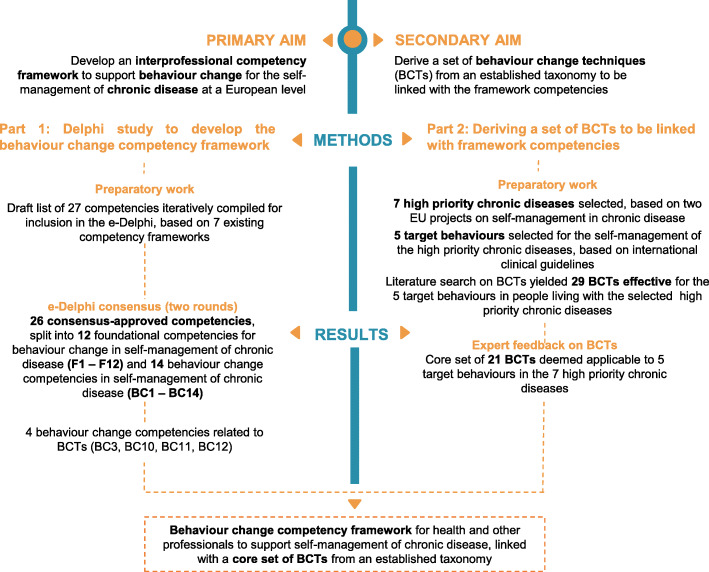
Overview of the development of the Train4Health competency framework and core set of linked behaviour change techniques

Figure 1 depicts the relationship between results of Parts 1 and 2, as presented in this section. It also illustrates the relationship between the primary and secondary aims, their respective methods, and the link between framework competencies and BCTs.

### Part 1: Delphi consensus on competencies

Sixty-one individuals responded to the invitation to participate in the Delphi study, of whom 55 agreed to receive the link to the questionnaire. Of the six individuals who declined the invitation, five cited a lack of relevant expertise and one cited a lack of time. Forty-eight individuals subsequently completed Round 1 of the Delphi study.

Panellists represented pharmacy (43.8%, *n* = 21), nursing (25%, *n* = 12) and sports sciences/physiotherapy (16.7%, *n* = 8), as well as a number of other disciplines (14.6%, *n* = 7) including general practice, nutrition, psychology and public health. Panellists originated from 12 European countries: Belgium (14.6%, *n* = 7), Estonia (2.1%, *n* = 1), Finland (2.1%, *n* = 1), Ireland (12.5%, *n* = 6), Lithuania (2.1%, *n* = 1), Malta (4.2%, *n* = 2), Netherlands (6.3%, *n* = 3), Norway (4.2%, *n* = 2), Portugal (20.8%, *n* = 10), Serbia (2.1%, *n* = 1), Spain (2.1%, *n* = 1), Switzerland (2.1%, *n* = 1), Turkey (4.2%, *n* = 2), UK (20.8%, *n* = 10).

**Fig. 2:**
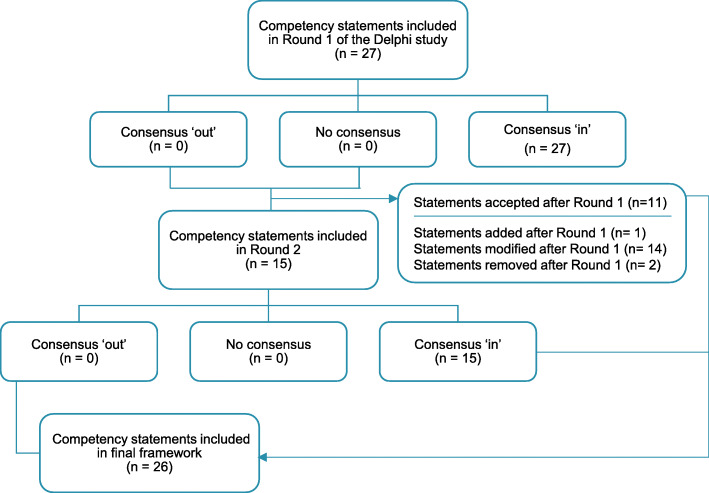
Overview of the progression of competency statements through the Delphi study

The overview of the Delphi results is depicted in Fig. 2. Consensus was achieved for all 27 competency statements in Round 1. Following a review of the panellists’ additional comments, the research team made the following amendments: two statements were removed, one statement was added, and 14 statements were modified (Table 3). The remaining eleven consensus-approved statements were not carried forward to Round 2. This round was therefore comprised of 15 statements.

**Table 3:** Examples of statements removed or modified after round 1

Statement	Round 1 ratings (median, IQR)	Example of panel member comments	Outcome
Ability to develop an intervention plan by selecting behaviour change techniques that are tailored to behaviour determinants and decide on their mode of delivery and content, depending on whether it is a brief or long-term intervention	4 (4–5)	*“In my opinion, this question should be split into two different questions because it is not the same to deliver a brief intervention (even being complex) or a long-term intervention)”* *“This is a complex item that entails different aspects - not easy to respond to - best revise for future rounds - perhaps split in 2 items”*	Statement split into two statements which were included in round 2: ● Ability to identify and select behaviour change techniques that are tailored to behavioural determinants (opportunities and barriers) in developing an intervention plan ● Ability to select behaviour change techniques that are appropriate to the length of the intervention (brief or long-term)
Ability to plan for addressing any other target behaviours that require attention	4 (3.75–4)	*“This is duplication of other competencies related to planning”*	Statement removed
Ability to screen for behavioural health factors e.g. use of substances, cognitive impairment, mental health	4 (4–5)	*“cognitive impairment is not a behavioural health factor nor is mental health in my modest opinion”*	Statement removed

The second round was completed by 40/48 panel members from Round 1. Lack of time was cited as the reason for non-participation by one individual and no reason was provided by the remaining individuals. Consensus was achieved for all 15 statements. This resulted in 26 statements being included in the final competency framework (Table 4). A complete summary of the progression of the competency statements through the Delphi study is provided in Additional file 4.

**Table 4:** The Train4Health competency framework v.1

Category	Statement
Foundational competencies for behaviour change in self-management of chronic disease	F1 Knowledge of the roles of other professionals in the local health system
	F2 Ability to maintain effective interprofessional relationships
	F3 Ability to provide interventions that are person-centred and consider the context (e.g. culture, family, local health system)
	F4 Ability to screen for readiness for behaviour change
	F5 Knowledge of the foundational aspects of effective communication
	F6 Ability to communicate effectively in partnership with people and families
	F7 Ability to communicate effectively with others (e.g. health care providers, administrators)
	F8 Ability to engage and partner with people individually and in groups
	F9 Ability to explore and manage expectations of individuals and groups
	F10 Knowledge of professional and ethical guidelines
	F11 Ability to demonstrate professional behaviour
	F12 Ability to reflect, self-evaluate and continuously develop these competencies
Behaviour change competencies in self-management of chronic disease	BC1 Knowledge of health behaviour and health beliefs
	BC2 Knowledge of appropriate behaviour change models/theories
	BC3 Knowledge of relevant behaviour change techniques
	BC4 Knowledge of clinical features of chronic diseases and target behaviours for their self-management
	BC5 Ability to identify self-management needs in relation to target behaviour(s) relevant for the chronic disease(s)
	BC6 Ability to engage and empower individuals with chronic diseases in self-management
	BC7 Ability to foster and maintain a good intervention alliance with individuals
	BC8 Ability to identify opportunities and barriers (determinants) to implementing change in the target behaviour
	BC9 Ability to work in partnership to prioritise target behaviours to develop an intervention plan
	BC10 Ability to identify and select behaviour change techniques that are tailored to behavioural determinants (opportunities and barriers) in developing an intervention plan
	BC11 Ability to select behaviour change techniques that are appropriate to the length of the intervention (brief or long-term)
	BC12 Ability to apply behaviour change techniques and implement the intervention plan, adapting and tailoring as required
	BC13 Ability to plan for follow-up and maintenance when the target behaviour has been achieved
	BC14 Ability to provide access to appropriate information and educational materials tailored to individual needs

### Part 2: expert feedback on BCTs

Table 5 provides an overview of the expert panel’s agreement on BCTs for the five target behaviours in the seven high priority chronic diseases considered. Agreement was not reached on applying two BCTs for “diet” in type 2 diabetes, obesity, cardiovascular and cardiometabolic diseases to the wider set of high priority chronic diseases considered, which included asthma and COPD; these were “2.5 Monitoring outcome(s) of behaviour by others without feedback” and “6.2 Social comparison”. The same happened regarding four BCTs in physical activity (in addition to the 2.5., previously mentioned, “3.1 Social support unspecified”, “10.4 Social reward” and “12.3 Avoidance/reducing exposure to cues for the behaviour” did not reach agreement). Additional BCTs suggested by at least two experts ranged from two for physical activity and 20 for medication adherence.

**Table 5:** Experts’ feedback on BCTs for the five target behaviours in seven high priority chronic diseases

	No. of evidence-based BCTs in persons living with either type 2 diabetes, obesity, cardiovascular or cardiometabolic diseases	No. of consensus-approved BCTs for the set of high priority chronic diseases considered	Additional BCTs suggested by at least two experts	Total number of BCTs
Diet (including alcohol intake)	21	19	7	26
Physical activity	27	23	2	25
Medication adherence	1	1	20	21
Smoking cessation	0	0	7	7
Symptom monitoring and management	0	0	9	9

Discussion within the research team led to a core set of 21 BCTs, common to the five target behaviours in the seven high priority chronic diseases considered (type 2 diabetes, COPD, obesity, heart failure asthma, hypertension and ischemic heart disease). Additional BCTs were organized in supplementary sets per target behaviour; both the core and supplementary lists of BCTs are presented in Additional file 5.

## Discussion

This study developed a behaviour change competency framework for health and other professionals to support behaviour change for the self-management of chronic disease. To authors’ knowledge this is the first interprofessional competency framework on the topic developed at European level. The framework comprises 26 competency statements, classified into two categories: foundational competencies for behaviour change in self-management of chronic disease and behaviour change competencies for self-management of chronic disease. These categories are similar to those outlined in a competency framework published by Dixon & Johnston [46], which provides mutual confirmation of their pertinence. In terms of content, the Train4Health framework clearly differentiates between competencies related to BCTs selection based on behaviour determinants (BC10) or the length of the intervention (BC11), and competencies related to BCTs application as part of an intervention plan (BC12). This approach is expected to facilitate competency assessment, as well as education and training.

There is a trend towards developing interprofessional frameworks for common competencies across health and other professions [47], such as the competencies needed to support behaviour change in people with chronic diseases. For example, the Irish initiative “Making every contact count” [48] and its British counterpart [49] draw on daily interactions with a variety of professions to support health behaviour change for preventing and managing chronic disease. The Train4Health competency framework statements are not profession-specific but interventions in some target behaviours may require knowledge and skills of a particular profession or group of professions. This will depend on case complexity, behaviour determinants and the person’s needs. For instance, an older person with multiple chronic conditions and complex needs may benefit from the knowledge and skills of an exercise physiologist, whilst promoting physical activity through an increase in walking in a person with less complex needs may be facilitated by any professional trained in relevant behaviour change interventions.

Twenty-six competency statements were consensually approved by panellists from several European regions and from a variety of disciplines. It is therefore reasonable to assume that the competency framework will be useful across Europe for a wide range of professions involved in behaviour change support for the self-management of chronic disease. Nonetheless, its European dimension will ultimately be determined by adoption beyond the five countries comprising the project strategic partnership (Portugal, Belgium, Ireland, Netherlands and Slovenia). One of the final Train4Health deliverables will be a White Paper with recommendations for large scale implementation of the educational package, combining lessons learnt during the project lifetime with findings from qualitative interviews with key stakeholders. This White Paper may also contribute to the adoption of the competency framework, given its intertwining nature with associated learning outcomes, curriculum and learning activities.

The overall number of competency statements obtained in the Train4Health framework is relatively small in comparison with some reference documents used as a starting point in preparatory work [13, 14] or the Dixon & Johnston competency framework recently published [46]. While lengthier competency frameworks may be credited with greater comprehensiveness, a predictable downside is ease of adoption and operationalisation. The fact that the Train4Health competency framework also serves to inform outcomes learners have to achieve allows higher granularity at this level and enables updates as evidence emerges, without necessarily having to change statements in the framework. Overall, there was a high level of agreement with individual competency statements subjected to panellists’ scrutiny and no comments pertaining to the structure of the competency framework, suggesting it was deemed appropriate. One reason that may explain this agreement is the fact that 25 statements were included in, or derivable from, three or more existing competency frameworks; this may be seen as an endorsement of its pertinence for behaviour change support. Similar to the seven existing frameworks reviewed as part of this study [7, 13–18], our competency framework does not endorse any particular behaviour change theory or model, thereby allowing for flexibility in the implementation of education and training, as well as in the delivery of the interventions. This in accordance with the rationale put forward in a NICE guideline, published in 2007 and still pertinent, stressing that the foci of training should be competencies and skills, rather than models of health behaviour and behaviour change [50].

Communication skills are unanimously recognised as pivotal in behaviour change support; Bull et al. neatly referred to them as the “how” of behaviour change, while BCTs comprise the “what” [51]. In the Train4Health competency framework, communication is encompassed in the foundational category of competencies (e.g. “F5 Knowledge of foundational aspects of effective communication”, “F6 Ability to communicate effectively in partnership with people and families”) whilst competencies that directly support behaviour change in the self-management of chronic disease draw on communication to “Engage and empower individuals with chronic diseases in self-management” (BC6) and “Foster and maintain a good intervention alliance” (BC7). It has been acknowledged that communication practices adopted by providers when supporting behaviour change have the potential to be detrimental. Recently, Albury et al. identified communications practices in health behaviour change associated with no response or minimal response [52]. For instance, initiating conversations by linking the person’s health concerns and their health behaviours often generated resistance displays [52]. Based on a systematic review and thematic synthesis of ten studies, targeting weight management (*n* = 5), smoking cessation (*n* = 3), safe sex (*n* = 2) and lowering alcohol consumption (*n* = 1), the authors of this systematic review also identified communication practices that facilitated the initiation and carrying out behaviour change conversations [52]. Attention has also turned to the importance of language in behaviour change support. Recommendations on preferred language to communicate with people living with obesity and diabetes have been co-produced with their involvement [53, 54]. An on-going systematic review on engaging older adults in self-management talk in healthcare encounters may also illuminate communication practices effective for behaviour change support in people living with chronic diseases [55]. Taken together, this body of knowledge represents a welcome contribution for training students and professionals in better communicating with people living with chronic diseases and step forward in developing competencies to engage and empower them.

There is no agreed method for developing competency frameworks. Common procedures are resorting to evidence, using existing frameworks as a starting point and collecting feedback from stakeholders [7, 13–18]. The Train4Health competency framework relied on these procedures and employed a scientific consensus method to collect stakeholders’ views. However, this approach is not without limitations. Firstly, a modification of the Delphi method was introduced, by providing the panel with numerical feedback but not the rating of each panellist. The reflection of panellists’ own rating in relation to the group’s is a hallmark of consensus building in the Delphi. That said, given the levels of agreement gauged in round 1, the impact of this modification appears minimal, if any. Consensus reflects experts’ opinion and should not be regarded as unconditional truth; the competency framework can iteratively evolve through its application in professional practice in Europe. Another limitation is the fact that persons living with chronic disease were not involved in the development of the competency framework, either through panel participation or via patient and public involvement, which raises doubts on what they find relevant in professionals’ competencies. This limitation can be partly mitigated by involving persons living with chronic disease in the dissemination, implementation and evaluation of research, as suggested by Shippee et al. [56].

The Train4Health competency framework is associated with a list of 21 core BCTs from an established taxonomy, yielded by a literature search in conjunction with expert feedback; the literature search can be replicated periodically to strengthen the evidence base of the BCTs set in people living with the selected chronic diseases. This list of standardised techniques to change behaviour, linked to competencies BC3, BC10, BC11 and BC12, is expected to enable a clearer description of behaviour change support in practice. Changing motivation, which is integral to the self-management of chronic disease, can be pursued by drawing on a recently published compendium of self-enactable techniques, such as “Emphasize autonomy”, “Find meaning in target behaviour” or “Self-monitoring of motivation” [57]. As part of future work these techniques could be linked to BC6 (“ability to engage and empower”) and be incorporated in the educational products.

It is noteworthy that identified target behaviours (e.g. diet, exercise, medication adherence) for the self-management of the high priority chronic diseases reflect essentially medical management [4]. This is unsurprising, considering that these self-management behaviours originated from clinical guidelines, reflecting what health professionals regard as important. Nonetheless, role management and emotional management may be equally, if not more important, to people living with chronic disease. This matter has been addressed through the formulation of learning outcomes related to BC6 “Ability to engage and empower individuals with chronic diseases in self-management”, focusing on the promotion of coping skills to manage the physical, emotional and social impacts of chronic disease in everyday life.

Using BCTs to train health or other professions is gaining acceptance, both in a research and practice context. The novelty of the Train4Health project is directing training to undergraduate students, facilitating future performance and reducing workforce challenges [58]. In the UK community pharmacists were trained to employ a set of 15 BCTs from BCTTv.1 to support non-adherent older persons with polypharmacy [59]. Interestingly, the list of 21 core BCTs obtained in the present study, which is applicable to medication adherence, has many commonalities with this work [59], in spite of resulting from a distinct method. Bull et al. used five BCTs from BCTTv.1 in a 2-day training intervention to upskill community health and social care practitioners in North East Scotland [51]. Course attendees (*n* = 156) cited healthy eating, smoking cessation, physical activity and medication adherence as common target behaviours discussed with the public in their practice. All BCTs used in this training initiative [51] are part of the set yielded in the present study, either as core (“1.4 Action planning”, “2.3 Self-monitoring of behaviour”, “5.1 Information about health consequences”) or supplementary BCTs (“7.1 Prompts and cues”, “9.2 Pros and cons”). In relation to these two studies, the Train4Health project presents a larger list of BCTs, applicable to five target behaviours. Ultimately, user testing of the Train4Health educational package (cases studies, massive open on-line course and a simulation software) to assess parameters such as usability and acceptability will shed light on whether this length is adequate for training purposes in an undergraduate population. Future intervention-based studies could also look to evaluate whether the competency framework facilitates improvements in professionals’ performance, as has been done with other competency frameworks for pharmacists [60].

It is likely that upskilling students to deliver these BCTs for five target behaviours – diet, physical activity, smoking cessation, medication adherence, symptoms managing and monitoring – in seven high priority chronic diseases will expand their competencies to additional target behaviours in the wider context of health behaviour change.

## Conclusions

The Train4Health competency framework and the accompanying set of standardized BCTs is a resource to health and other professionals across Europe, as well as workforce regulators, to supplement existing frameworks in respect to behaviour change in chronic disease, set standards and guide training. Since EU countries recognise each other’s professional qualifications, a shared competency framework is therefore of considerable value. The competency framework is equally important to guide the development of a learning outcomes-based curriculum in behaviour change support in chronic disease and drive the development of educational products for undergraduate students in several disciplines.

## Supplementary Information

**Additional file 1.** Merged long-list of competencies based on [7, 13–18].

**Additional file 2.** T4H Round 1 Delphi questionnaire.

**Additional file 3.** Glossary.

**Additional file 4.** Summary of progression of the competency statements through the Delphi.

**Additional file 5: Table 1.** Core set of behaviour change techniques (BCTs) applicable to five target behaviours in seven high priority chronic diseases. **Table 2.** Supplementary BCTs per target behaviours in seven high priority chronic diseases.

## Data Availability

The dataset is available from the corresponding author on reasonable request. Supplementary materials will be available in the project Open Science Framework repository.

## Abbreviation

BCTs: Behaviour Change Techniques
