# Supplementary material for: Development of a European competency framework for health and other professionals to support behaviour change in persons self-managing chronic disease
Source: BMC Med Educ. 2021 May 20;21:287. doi: 10.1186/s12909-021-02720-w (PMC8136137; doi:10.1186/s12909-021-02720-w)
Supplement: Supplementary file 1 — Additional file 1. Merged long-list of competencies based on [7, 13–18]. [file 12909_2021_2720_MOESM1_ESM.docx]

**Development of a European competency framework for health and other professionals to support behaviour change in persons** **self-managing** **chronic disease**

Mara Pereira Guerreiro^1, 2^, Judith Strawbridge^3^, Afonso Miguel Cavaco^4^, Isa Brito Félix^1^, Marta Moreira Marques^5^, Cathal Cadogan^6^

^1^ CIDNUR, Nursing School of Lisbon, Lisbon, Portugal

^2^ CiiEM, Instituto Universitário Egas Moniz, Monte de Caparica, Portugal

^3^ School of Pharmacy and Biomolecular Sciences, Royal College of Surgeons in Ireland, Dublin, Ireland

^4^ Faculty of Pharmacy, University of Lisbon, Lisbon, Portugal

^5^ Trinity College Dublin; ADAPT SFI Research Centre & Trinity Centre for Practice and Healthcare Innovation, Dublin

^6^ School of Pharmacy and Pharmaceutical Sciences, Trinity College Dublin, Dublin, Ireland

**Corresponding author:**

Dr. Mara Pereira Guerreiro

mara.guerreiro@esel.pt

**Additional file 1**

**Merged long-list of competencies based on [7, 13–18]**

|  | **Behaviour change techniques that directly support self-management of chronic disease** |
| --- | --- |
|  | *Knowledge of* |
| 1 | Clinical features of chronic diseases and their management |
| 2 | Health behaviour and health beliefs |
| 3 | Appropriate behaviour change models/theories |
| 4 | Key behaviour change techniques |
| 5 | Self-monitoring |
|  |  |
|  | *Ability to* |
| 6 | Engage and activate patients in self-management |
| 7 | Foster and maintain a good intervention alliance |
| 8 | Recognise opportunities and barriers to implementing interventions |
| 9 | Agree goals (behaviours and outcome) |
| 10 | Make action plans based on identified goals |
| 11 | Create and implement care plans that use the most appropriate intervention method to address behavioural health factors |
| 12 | Provide a range of (brief and longer-term) interventions effectively |
| 13 | Implement behaviour change in a manner consistent with its underlying philosophy (problem solve, maintain flexibility) |
| 14 | Use measures and self-monitoring to guide interventions and outcomes |
| 15 | Respond to feedback and adapt accordingly |
| 16 | Carry out health behaviour problem-solving |
| 17 | End interventions and plan for long-term maintenance |
| 18 | Support access to appropriate information and educational materials |
|  |  |
|  | **Adjuvant activities that indirectly support self-management of chronic disease** |
|  | *Ability to* |
| 19 | Address behavioural health factors including mental health, substance use etc. |
| 20 | Give advice about additional resources and support |
|  |  |
|  | **Additional competencies required for effective delivery of specific behaviour change and or adjunct activities** |
|  | **Intervention delivery** |
|  | *Knowledge of* |
| 21 | The potential significance and impact of social and cultural differences on the effectiveness and acceptability of interventions |
|  |  |
|  | *Ability to* |
| 22 | Forward plan for an effective intervention |
| 23 | Work as part of an interprofessional team |
| 24 | Provide culturally responsive, whole person and family orientated care |
| 25 | Appropriately follow up care |
|  |  |
|  | **Information gathering** |
|  | *Ability to* |
| 26 | Identify and assess behavioural health needs |
| 27 | Screen for behavioural health factors e.g. use of substances, cognitive impairment, mental health |
| 28 | Screen for readiness and suitability for behaviour change |
|  |  |
|  | **General communication** |
|  | *Knowledge of* |
| 29 | The principles of good communication skills |
|  |  |
|  | *Ability to* |
| 30 | Communicate effectively with patients (establish rapport, build patient understanding, question appropriately, actively listen, jargon-free delivery, non-judgemental, appropriately paced, empathetic, in partnership) |
| 31 | Communicate effectively with others (families, health care providers) |
| 32 | Work with groups |
| 33 | Manage expectations |
|  |  |
|  | **Professionalism** |
|  | *Knowledge of* |
| 34 | Professional and ethical guidelines |
| 35 | The roles of the other team members |
|  |  |
|  | *Ability to* |
| 36 | Demonstrate professional behaviour |
| 37 | Make use of supervision |
| 38 | Uphold equality |
| 39 | Create a safe environment |
| 40 | Reflect |
| 41 | Work as an effective interprofessional team member, inclusive of patients and families |
| 42 | Value the members of the interprofessional team |
| 43 | Resolve conflicts |
| 44 | Plan and manage the service (procedures, protocols, entrepreneurship) |
| 45 | Evaluate the service (outcome measures) |
| 46 | Develop and innovate the service |
| 47 | Conduct research |

7. Vallis M, Lee-Baggley D, Sampalli T, Ryer A, Ryan-Carson S, Kumanan K, et al. Equipping providers with principles, knowledge and skills to successfully integrate behaviour change counselling into practice: a primary healthcare framework. Public Health. 2017;154:70–8.

13. Dixon D, Johnston M. Health Behaviour Change Competency Framework: Competences to deliver interventions to change lifestyle behaviours that affect health. 2010. http://www.healthscotland.com/documents/4877.aspx

14. Payne K, De Normanville C, Stansfield K, Barnnet N, Machaczek K, Qutishat D, et al. Prevention and Lifestyle Behaviour Change A Competence Framework. 2010. ﻿https://www.makingeverycontactcount.co.uk/media/1017/011-prevention-and-lifestyle-behaviour-change-a-competence-framework.pdf

15. de Jong J, Dikkeboer B, Bruining C. European Bachelor Physical Activity and Lifestyle (PAL). 2013.

16. Hoge MA, Morris JA, Laraia M, Pomerantz A, Farley T. Core Competencies for Integrated Behavioral Health and Primary Care. 2014. ﻿www.integration.samhsa.gov

17. Health Behaviour Change and Education Development Working Group. Core Competences for the Health Behaviour Change and Education Component for Cardiovascular Disease Prevention and Rehabilitation Services. 2016. ﻿http://www.bacpr.com/images/BACPR Core Competences for the Health Behaviour Change and Education Component 2016.pdf

18. Miller BF, Gilchrist EC, Ross KM, Wong SL, Blount A, Peek CJ. Core Competencies for Behavioral Health Providers Working in Primary Care. Prepared from the Colorado Consensus Conference. 2016. ﻿https://www.google.com/url?sa=t&rct=j&q=&esrc=s&source=web&cd=&ved=2ahUKEwjf8-iR7ZnwAhUUjhQKHXOEARkQFjAAegQIBBAD&url=https%3A%2F%2Fwww.rmhp.org%2F-%2Fmedia%2FRMHPdotOrg%2FFiles%2FPDF%2FProvider%2FPractice-Transformation-Programs%2FCO-EARTH-RESOURCES%2FBasics-of-Integration%2FCore-Competencies-BHP.ashx&usg=AOvVaw13288w9AC9VXRSMRf6muLi
