# Supplementary material for: Development of a European competency framework for health and other professionals to support behaviour change in persons self-managing chronic disease
Source: BMC Med Educ. 2021 May 20;21:287. doi: 10.1186/s12909-021-02720-w (PMC8136137; doi:10.1186/s12909-021-02720-w)
Supplement: Supplementary file 2 — Additional file 2. T4H Round 1 Delphi questionnaire. [file 12909_2021_2720_MOESM2_ESM.docx]

**Development of a European competency framework for health and other professionals to support behaviour change in persons** **self-managing** **chronic disease**

Mara Pereira Guerreiro^1, 2^, Judith Strawbridge^3^, Afonso Miguel Cavaco^4^, Isa Brito Félix^1^, Marta Moreira Marques^5^, Cathal Cadogan^6^

^1^ CIDNUR, Nursing School of Lisbon, Lisbon, Portugal

^2^ CiiEM, Instituto Universitário Egas Moniz, Monte de Caparica, Portugal

^3^ School of Pharmacy and Biomolecular Sciences, Royal College of Surgeons in Ireland, Dublin, Ireland

^4^ Faculty of Pharmacy, University of Lisbon, Lisbon, Portugal

^5^ Trinity College Dublin; ADAPT SFI Research Centre & Trinity Centre for Practice and Healthcare Innovation, Dublin

^6^ School of Pharmacy and Pharmaceutical Sciences, Trinity College Dublin, Dublin, Ireland

**Corresponding author:**

Dr. Mara Pereira Guerreiro

mara.guerreiro@esel.pt

**Additional file 2**

**T4H Round 1 Delphi questionnaire**

Thank you taking the time to participate in this study. The aim of this study is to develop a European competency framework to train health professions students to support behaviour change and self-management in chronic disease. The purpose of the Delphi exercise is to reach consensus (agreement) with regard to the competencies to include in this framework.

The questionnaire will take approximately 15 minutes to complete.

Please enter the ID code given to you in the study invitation letter: [insert ID code]

General Information

1. What gender do you identify as?
2. Male
3. Female
4. Non-binary
5. Prefer not to answer
6. What is your profession? Please select one of the following.
7. Nurse
8. Exercise physiologist
9. Pharmacist
10. Other – [text box]
11. What country are you based in?

[dropdown list]

**Instructions on how to complete the questionnaire**

We are interested in your views on the competencies listed below for inclusion in a European competency framework to train health professions students from pharmacy, nursing and sports sciences to support behaviour change and self-management in chronic disease

Within the questionnaire, you will be asked to score how important you think each competency is using a 5-point scale (where 1 indicates strongly disagree and 5 indicates strongly agree). The final question will ask you to suggest any additional competencies that you think might be important but have not been presented in the main questionnaire.

Consensus (agreement) will be achieved when ≥75% of panel members agree that a competency should be included in the in the final list.

**Competency statements**

Please rate your level of agreement with each competency from 1 to 5, using the scale below. Alternatively, you can state if you are unsure about a particular competency.

1. Strongly disagree
2. Disagree
3. Neither agree or disagree
4. Agree
5. Strongly agree

**Category 1: Competency statements that directly support behaviour change in the self-management of chronic disease**

1. Knowledge of health behaviour and health beliefs
2. Knowledge of appropriate behaviour change models/theories
3. Knowledge of relevant behaviour change techniques
4. Knowledge of clinical features of chronic diseases and target behaviours for their self-management
5. Ability to identify self-management needs in relation to target behaviour(s) relevant for the chronic disease(s)
6. Ability to engage and activate individuals with chronic diseases in self-management
7. Ability to foster and maintain a good intervention alliance
8. Ability to identify opportunities and barriers to implementing behaviour change interventions
9. Ability to prioritise target behaviours
10. Ability to develop an intervention plan by selecting behaviour change techniques that are tailored to behaviour determinants and decide on their mode of delivery and content, depending on whether it is a brief or long-term intervention
11. Ability to effectively implement the plan developed for the brief or long-term intervention
12. Ability to plan for follow-up and maintenance when the target behaviour has been achieved
13. Ability to provide access to appropriate information and educational materials
14. Ability to plan for addressing any other target behaviours that require attention

**Category 2: Foundational competencies required for effective delivery of behaviour change support**

1. Knowledge of the roles of the other team members
2. Ability to work as part of an interprofessional team
3. Ability to provide culturally responsive, whole person and family-orientated interventions
4. Ability to screen for behavioural health factors e.g. use of substances, cognitive impairment, mental health
5. Ability to screen for readiness and suitability for behaviour change
6. Knowledge of the foundational aspects of effective communication
7. Ability to communicate effectively in partnership with people and families
8. Ability to communicate effectively with others (e.g. health care providers, administrators)
9. Ability to work with patient groups
10. Ability to manage expectations
11. Knowledge of professional and ethical guidelines
12. Ability to demonstrate professional behaviour
13. Ability to reflect and evaluate one's own support to continuously develop these competencies

[NOTE: free-text response boxes were included after each competency statement to allow participants to comment on any of the statements.]

**Additional comments**

Do you have any additional comments on the proposed framework or suggestions for items to be included?

[free text response]
