# Supplementary material for: Development of a European competency framework for health and other professionals to support behaviour change in persons self-managing chronic disease
Source: BMC Med Educ. 2021 May 20;21:287. doi: 10.1186/s12909-021-02720-w (PMC8136137; doi:10.1186/s12909-021-02720-w)
Supplement: Supplementary file 3 — Additional file 3. Glossary. [file 12909_2021_2720_MOESM3_ESM.docx]

**Development of a European competency framework for health and other professionals to support behaviour change in persons** **self-managing** **chronic disease**

Mara Pereira Guerreiro^1, 2^, Judith Strawbridge^3^, Afonso Miguel Cavaco^4^, Isa Brito Félix^1^, Marta Moreira Marques^5^, Cathal Cadogan^6^

^1^ CIDNUR, Nursing School of Lisbon, Lisbon, Portugal

^2^ CiiEM, Instituto Universitário Egas Moniz, Monte de Caparica, Portugal

^3^ School of Pharmacy and Biomolecular Sciences, Royal College of Surgeons in Ireland, Dublin, Ireland

^4^ Faculty of Pharmacy, University of Lisbon, Lisbon, Portugal

^5^ Trinity College Dublin; ADAPT SFI Research Centre & Trinity Centre for Practice and Healthcare Innovation, Dublin

^6^ School of Pharmacy and Pharmaceutical Sciences, Trinity College Dublin, Dublin, Ireland

**Corresponding author:**

Dr. Mara Pereira Guerreiro

mara.guerreiro@esel.pt

**Additional file 3**

**Glossary**

| Behaviour | Anything a person does in response to internal or external events. Actions may be overt (motor or verbal) and directly measurable or, covert (activities not viewable but involving voluntary muscles) and indirectly measurable; behaviours are physical events that occur in the body and are controlled by the brain (Davis, Campbell, Hildon, Hobbs, & Michie, 2015). |
| --- | --- |
| Behaviour change | Refers to any transformation or modification of human behaviour. |
| Behaviour change intervention | An intervention that has the aim of influencing human behaviour (Michie et al 2020). |
| Behaviour change competency framework | Describes the knowledge and skills required to deliver interventions to people to help them change their behaviour (Dixon and Johnston, 2010). |
| Behaviour change model/theory | A set of concepts and/or statements which specify how phenomena relate to each other, providing an organizing description of a system that accounts for what is known, and explains and predicts phenomena (Davis, Campbell, Hildon, Hobbs, & Michie, 2015). In the context of behaviour change, theories seek to explain why, when and how a behaviour does or does not occur, and identify sources of influence to be targeted in order to alter behaviour. |
| Behaviour change support education (behavioural support education) | An activity or coordinated set of activities provided to healthcare professionals or students to acquire knowledge and skills to support behaviour change in health. |
| Behaviour change support in health | An activity or coordinated set of activities provided by a healthcare professional that aims to get an individual to behave differently from how she or he would have acted without such an action (adapted from NICE, 2014) |
| Behaviour change technique (BCT) | An observable, replicable, an irreducible component of an intervention designed to change behaviour and a postulated active ingredient within the intervention (Michie, Atkins, & West, 2014). For examples, please refer to the document on core BCTs. |
| Behavioural determinants | Factors that influence the behaviour either in a positive or a negative way. |
| Brief intervention | Intervention delivered in a short interaction between the provider and the individual, often carried out when the opportunity arises, typically taking no more than a few minutes. Although short in duration, a brief intervention can be delivered in several sessions (adapted from NICE, 2014). |
| Chronic disease | Disease that persists over a long period of time and generally has slow progression, requiring ongoing attention from healthcare professionals. Cardiovascular diseases (e.g. heart failure and stroke), cancers, respiratory diseases (such as chronic obstructive pulmonary disease and asthma) and diabetes are the most common chronic diseases (WHO, 2016) |
| Content | A planned process that is part of a behaviour change intervention and is intended to be causally active in influencing the outcome behaviour (Michie et al, 2020). |
| Empower | Involves equipping patients (and their informal caregivers whenever appropriate) with the capacity to participate in decisions related to their condition to the extent that they wish to do so; to become “co-managers” of their condition in partnership with health professionals; and to develop self-confidence, self-esteem and coping skills to manage the physical, emotional and social impacts of illness in everyday life (EMPATHiE 2014). |
| Health behaviour | Any behaviour that a person engages in that can affect their health in either a positive or negative way (British Association for Cardiovascular Prevention and Rehabilitation, 2016) |
| Intervention alliance | Refers to a co-operative working relationship between the patient and their healthcare professional |
| Long-term intervention | Intervention delivered in a longer interaction (e.g. around 30 minutes) between the provider and the individual, which has a structured plan, and consists of multiple sessions over time (adapted from NICE, 2014). |
| Mode of delivery | A part of a behaviour change intervention that is the means by which the content (e.g. BCT) is provided. For example, face-to-face is a mode of delivery involving an intervention source and recipient being together in the same location and communicating directly (Marques et al., 2020). |
| Self-management | Defined as tasks performed by an individual to minimize the impact of one’s disease, with or without the support of health professionals. Tasks can holistically be categorized under medical management (e.g. taking medication, adhering to a diet, engaging in physical activity), role management (e.g. redefining life roles in light of a chronic disease) and emotional management (e.g. dealing with anger and frustration) and are related to a set of skills (adapted from Lorig & Holman, 2003 and PRO-STEP, 2018). |

# References

British Association for Cardiovascular Prevention and Rehabilitation. (2016). *Core Competences for the Health Behaviour Change and Education Component for Cardiovascular Disease Prevention and Rehabilitation Services*. London.

Davis, R., Campbell, R., Hildon, Z., Hobbs, L., & Michie, S. (2015). Theories of behaviour and behaviour change across the social and behavioural sciences: a scoping review. *Health Psychology Review*, *9*(3), 323–344. https://doi.org/http://dx.doi.org/10.1080/17437199.2014.941722

Dixon, D. & Johnston, M. (2010). Health Behaviour Change Competency Framework: Competences to deliver interventions to change lifestyle behaviours that affect health. BPS.

EMPATHiE: Empowering patients in the management of chronic diseases. FINAL SUMMARY REPORT. 2014

Lorig KR, Holman H. Self-management education: history, definition, outcomes, and mechanisms. *Ann Behav Med*. 2003;26(1):1‐7. doi:10.1207/S15324796ABM2601_01

Marques M.M, Carey R., Norris E., Evans F., Finnerty A.N., Hastings J., Jenkins E., Johnston M., West R., & Michie S. Delivering Behaviour Change Interventions: Development of a Mode of Delivery Ontology. *Wellcome Open Research* (Accepted).

Michie S., West R., Finnerty A.N., Norris E., Wright A.J., Marques M.M., Johnston M., Kelly M.P., Thomas J. Hastings J. Representation of behaviour change interventions and their evaluation: Development of the Upper Level of the Behaviour Change Intervention Ontology. *Wellcome Open Research* (Accepted)

Michie, S., Atkins, L., & West, R. (2014). *The Behavior Change Wheel: A Guide To Designing Interventions* (First edit). London: Silverback Publishing.

National Institute for Health and Care Excellence. (2014). *NICE Guidance: Behaviour change: individual approaches*. Retrieved from <https://www.nice.org.uk/guidance/ph49>

PRO-STEP Project Consortium. (2018). *Promoting Self-management for chronic diseases in Europe Pilot Project on the Promotion of Self-Care in Chronic Diseases in the European Union*.

WHO (2016). Noncommunicable Diseases. Available from <http://www.who.int/topics/noncommunicable_diseases/en/>
