# Supplementary material for: Development of a European competency framework for health and other professionals to support behaviour change in persons self-managing chronic disease
Source: BMC Med Educ. 2021 May 20;21:287. doi: 10.1186/s12909-021-02720-w (PMC8136137; doi:10.1186/s12909-021-02720-w)
Supplement: Supplementary file 4 — Additional file 4. Summary of progression of the competency statements through the Delphi. [file 12909_2021_2720_MOESM4_ESM.docx]

**Development of a European competency framework for health and other professionals to support behaviour change in persons** **self-managing** **chronic disease**

Mara Pereira Guerreiro^1, 2^, Judith Strawbridge^3^, Afonso Miguel Cavaco^4^, Isa Brito Félix^1^, Marta Moreira Marques^5^, Cathal Cadogan^6^

^1^ CIDNUR, Nursing School of Lisbon, Lisbon, Portugal

^2^ CiiEM, Instituto Universitário Egas Moniz, Monte de Caparica, Portugal

^3^ School of Pharmacy and Biomolecular Sciences, Royal College of Surgeons in Ireland, Dublin, Ireland

^4^ Faculty of Pharmacy, University of Lisbon, Lisbon, Portugal

^5^ Trinity College Dublin; ADAPT SFI Research Centre & Trinity Centre for Practice and Healthcare Innovation, Dublin

^6^ School of Pharmacy and Pharmaceutical Sciences, Trinity College Dublin, Dublin, Ireland

**Corresponding author:**

Dr. Mara Pereira Guerreiro

mara.guerreiro@esel.pt

**Additional file 4**

Summary of progression of the competency statements through the Delphi

| **Statement** | **Round 1 ratings (median, IQR)** | **Outcome** | **Revised statement** | **Round 2 ratings (median, IQR)** | **Outcome** |
| --- | --- | --- | --- | --- | --- |
| 1. Knowledge of health behaviour and health beliefs | 5 (4 -5) | Accept | N/A | N/A | N/A |
| 1. Knowledge of appropriate behaviour change models/theories | 4 (4 -5) | Accept | N/A | N/A | N/A |
| 1. Knowledge of relevant behaviour change techniques | 5 (4 -5) | Accept | N/A | N/A | N/A |
| 1. Knowledge of clinical features of chronic diseases and target behaviours for their self-management | 4 (4 -5) | Accept | N/A | N/A | N/A |
| 1. Ability to identify self-management needs in relation to target behaviour(s) relevant for the chronic disease(s) | 5 (4 -5) | Accept | N/A | N/A | N/A |
| 1. Ability to engage and activate individuals with chronic diseases in self-management | 5 (4 -5) | Revise based on additional comments | Ability to engage and empower individuals with chronic diseases in self-management | 5 (4.75 -5) | Accept |
| 1. Ability to foster and maintain a good intervention alliance | 4 (4 -5) | Revise based on additional comments | Ability to foster and maintain a good intervention alliance with individuals | 4 (4 -5) | Accept |
| 1. Ability to identify opportunities and barriers to implementing behaviour change interventions | 5 (4 -5) | Revise based on additional comments | Ability to identify opportunities and barriers (determinants) to implementing change in the target behaviour | 5 (4 -5) | Accept |
| 1. Ability to prioritise target behaviours to develop an intervention plan | 4 (4 -5) | Revise based on additional | Ability to work in partnership to prioritise target behaviours to develop an intervention plan | 5 (4 -5) | Accept |
| 1. Ability to develop an intervention plan by selecting behaviour change techniques that are tailored to behaviour determinants and decide on their mode of delivery and content, depending on whether it is a brief or long-term intervention | 4 (4 -5) | Revise based on additional comments | Ability to identify and select behaviour change techniques that are tailored to behavioural determinants (opportunities and barriers) in developing an intervention plan | 4 (4 -5) | Accept |
|  |  |  | New statement: Ability to select behaviour change techniques that are appropriate to the length of the intervention (brief or long-term) | 4 (4 -5) | Accept |
| 1. Ability to effectively implement the plan developed for the  brief or long-term intervention | 4 (4 -5) | Revise based on additional comments | Ability to apply behaviour change techniques and implement the intervention plan, adapting and tailoring as required | 4 (4 -5) | Accept |
| 1. Ability to plan for follow-up and maintenance when the target behaviour has been achieved | 4 (4 -5) | Accept | N/A | N/A | N/A |
| 1. Ability to provide access to appropriate information and educational materials | 4 (4 -5) | Revise based on additional comments | Ability to provide access to appropriate information and educational materials tailored to individual needs | 5 (4 -5) | Accept |
| 1. Ability to plan for addressing any other target behaviours that require attention | 4 (4 -5) | Removed based on additional comments | N/A | N/A | N/A |
| 1. Knowledge of the roles of the other team members | 4 (4 -5) | Revise based on additional comments | Knowledge of the roles of other professionals in the local health system | 4 (4 -5) | Accept |
| 1. Ability to work as part of an interprofessional team | 5 (4 -5) | Revise based on additional comments | Ability to maintain effective interprofessional relationships | 5 (4 -5) | Accept |
| 1. Ability to provide culturally responsive, whole person and family-orientated interventions | 5 (4 -5) | Revise based on additional comments | Ability to provide interventions that are person-centred and consider the context (e.g. culture, family, local health system) | 5 (5 -5) | Accept |
| 1. Ability to screen for behavioural health factors e.g. use of substances, cognitive impairment, mental health | 4 (4 -5) | Removed based on additional comments | N/A | N/A | N/A |
| 1. Ability to screen for readiness and suitability for behaviour change | 4 (4 -5) | Revise based on additional comments | Ability to screen for readiness for behaviour change | 4 (4 -5) | Accept |
| 1. Knowledge of the foundational aspects of effective communication | 5 (4 -5) | Accept | N/A | N/A | N/A |
| 1. Ability to communicate effectively in partnership with people and families | 5 (5 -5) | Accept | N/A | N/A | N/A |
| 1. Ability to communicate effectively with others (e.g. health care providers, administrators) | 5 (4 -5) | Accept | N/A | N/A | N/A |
| 1. Ability to work with patient groups | 4 (4 -5) | Revise based on additional comments | Ability to engage and partner with people individually and in groups | 4 (4 -5) | Accept |
| 1. Ability to manage expectations | 4 (4 -5) | Revise based on additional comments | Ability to explore and manage expectations of individuals and groups | 4 (4 -5) | Accept |
| 1. Knowledge of professional and ethical guidelines | 5 (4 -5) | Accept | N/A | N/A | N/A |
| 1. Ability to demonstrate professional behavior | 5 (4 -5) | Accept | N/A | N/A | N/A |
| 1. Ability to reflect and evaluate one's own support to continuously develop these competencies | 5 (4 -5) | Revise based on additional comments | Ability to reflect, self-evaluate and continuously develop these competencies | 5 (4 -5) | Accept |
