# Supplementary material for: Development of a European competency framework for health and other professionals to support behaviour change in persons self-managing chronic disease
Source: BMC Med Educ. 2021 May 20;21:287. doi: 10.1186/s12909-021-02720-w (PMC8136137; doi:10.1186/s12909-021-02720-w)
Supplement: Supplementary file 5 — Additional file 5: Table 1. Core set of behaviour change techniques (BCTs) applicable to five target behaviours in seven high priority chronic diseases. Table 2. Supplementary BCTs per target behaviours in seven high priority chronic diseases. [file 12909_2021_2720_MOESM5_ESM.docx]

**Development of a European competency framework for health and other professionals to support behaviour change in persons** **self-managing** **chronic disease**

Mara Pereira Guerreiro^1, 2^, Judith Strawbridge^3^, Afonso Miguel Cavaco^4^, Isa Brito Félix^1^, Marta Moreira Marques^5^, Cathal Cadogan^6^

^1^ CIDNUR, Nursing School of Lisbon, Lisbon, Portugal

^2^ CiiEM, Instituto Universitário Egas Moniz, Monte de Caparica, Portugal

^3^ School of Pharmacy and Biomolecular Sciences, Royal College of Surgeons in Ireland, Dublin, Ireland

^4^ Faculty of Pharmacy, University of Lisbon, Lisbon, Portugal

^5^ Trinity College Dublin; ADAPT SFI Research Centre & Trinity Centre for Practice and Healthcare Innovation, Dublin

^6^ School of Pharmacy and Pharmaceutical Sciences, Trinity College Dublin, Dublin, Ireland

**Corresponding author:**

Dr. Mara Pereira Guerreiro

mara.guerreiro@esel.pt

**Additional file 5**

**Table 1: Core set of behaviour change techniques (BCTs) applicable to five target behaviours in seven high priority chronic diseases**

| **BCT** | **Definition (Michie et al., 2013)** |
| --- | --- |
| 1.1 Goal setting (behaviour) | Set or agree on a goal defined in terms of the behavior to be achieved |
| 1.2 Problem solving | Analyse, or prompt the person to analyse, factors influencing the behavior and generate or select strategies that include overcoming barriers and/or increasing facilitators |
| 1.3 Goal setting (outcomes) | Set or agree on a goal defined in terms of a positive outcome of wanted behavior |
| 1.4 Action planning | Prompt detailed planning of performance of the behavior (must include at least one of context, frequency, duration and intensity). Context may be environmental (physical or social) or internal (physical, emotional or cognitive) (includes ‘Implementation Intentions’) |
| 1.5 Review behaviour goal(s) | Review behavior goal(s) jointly with the person and consider modifying goal(s) or behavior change strategy in light of achievement. This may lead to re-setting the same goal, a small change in that goal or setting a new goal instead of (or in addition to) the first, or no change |
| 1.6 Discrepancy between current behaviour and goal | Draw attention to discrepancies between a person’s current behavior (in terms of the form, frequency, duration, or intensity of that behavior) and the person’s previously set outcome goals, behavioral goals or action plans (goes beyond self-monitoring of behavior) |
| 1.7 Review outcomes goal(s) | Review outcome goal(s) jointly with the person and consider modifying goal(s) in light of achievement. This may lead to resetting the same goal, a small change in that goal or setting a new goal instead of, or in addition to the first |
| 2.2 Feedback on behaviour | Monitor and provide informative or evaluative feedback on performance of the behavior (e.g. form, frequency,  duration, intensity) |
| 2.3 Self-monitoring of behaviour | Establish a method for the person to monitor and record their behavior(s) as part of a behavior change strategy |
| 2.4 Self-monitoring of outcome(s) of behaviour | Establish a method for the person to monitor and record the outcome(s) of their behavior as part of a behavior change strategy |
| 2.6 Biofeedback | Provide feedback about the body (e.g. physiological or biochemical state) using an external monitoring device as part of a behavior change strategy |
| 2.7 Feedback on outcome(s) of behaviour | Monitor and provide feedback on the outcome of performance of the behavior |
| 3.1 Social support (unspecified) | Advise on, arrange or provide social support (e.g. from friends, relatives, colleagues,’ buddies’ or staff) or noncontingent praise or reward for performance of the behavior. It includes encouragement and counselling, but only when it is directed at the behavior |
| 3.2 Social support (practical) (4) | Advise on, arrange, or provide practical help (e.g. from friends, relatives, colleagues, ‘buddies’ or staff) for performance of the behavior |
| 3.3 Social support (emotional) (4) | Advise on, arrange, or provide emotional social support (e.g. from friends, relatives, colleagues, ‘buddies’ or staff) for performance of the behavior |
| 4.1 Instruction on how to perform a behaviour | Advise or agree on how to perform the behavior (includes ‘Skills training’) |
| 5.1 Information about health consequences | Provide information (e.g. written, verbal, visual) about health consequences of performing the behavior |
| 8.3 Habit formation (2) | Prompt rehearsal and repetition of the behavior in the same context repeatedly so that the context elicits the behavior |
| 11.2 Reduce negative emotions (4) | Advise on ways of reducing negative emotions to facilitate performance of the behavior (includes ‘Stress Management’) |
| 12.1 Restructuring the physical environment (2) | Change, or advise to change the physical environment in order to facilitate performance of the wanted behavior or create barriers to the unwanted behavior (other than prompts/cues, rewards and punishments) |
| 12.5 Adding objects to the environment | Add objects to the environment in order to facilitate performance of the behavior |

Michie, S., Richardson, M., Johnston, M., Abraham, C., Francis, J., Hardeman, W., Eccles, M. P., Cane, J., & Wood, C. E. (2013). The behavior change technique taxonomy (v1) of 93 hierarchically clustered techniques: Building an international consensus for the reporting of behavior change interventions. *Annals of Behavioral Medicine*, *46*(1), 81–95. https://doi.org/10.1007/s12160-013-9486-6

**Table 2: Supplementary BCTs per target behaviours in seven high priority chronic diseases**

| **Diet** | **Physical activity** | **Medication adherence** | **Smoking cessation** | **Symptom monitoring and management** | **BCT definition (Michie et al., 2013)** |
| --- | --- | --- | --- | --- | --- |
| 6.1 Demonstration of the behaviour | 6.1 Demonstration of the behaviour |  |  |  | Provide an observable sample of the performance of the behaviour, directly in person or indirectly e.g. via film, pictures, for the person to aspire to or imitate (includes ‘Modelling’). |
| 7.1 Prompts/cues | 7.1 Prompts/cues | 7.1 Prompts/cues |  | 7.1 Prompts/cues | Introduce or define environmental or social stimulus with the purpose of prompting or cueing the behavior. The prompt or cue would normally occur at the time or place of performance |
| 8.1 Behavioural practice/rehearsal | 8.1 Behavioural practice/rehearsal | 8.1 Behavioural practice/rehearsal |  |  | Prompt practice or rehearsal of the performance of the behavior one or more times in a context or at a time when the performance may not be necessary, in order to increase habit and skill |
| 8.7 Graded tasks | 8.7 Graded tasks | 8.7 Graded tasks |  |  | Set easy-to-perform tasks, making them increasingly difficult, but achievable, until behavior is performed |
| 9.2 Pros and cons | 9.2 Pros and cons | 9.2 Pros and cons | 9.2 Pros and cons |  | Advise the person to identify and compare reasons for wanting (pros) and not wanting to (cons) change the behavior (includes ‘Decisional balance’) |
|  | 11.1 Pharmacological support |  |  |  | Provide, or encourage the use of or adherence to, drugs to facilitate behavior change |
| 12.3 Avoidance/reducing exposure to cues for the behaviour |  |  | 12.3 Avoidance/reducing exposure to cues for the behaviour |  | Advise on how to avoid exposure to specific social and contextual/physical cues for the behavior, including changing daily or weekly routines |
|  | 13.1 Identification of self as role model |  |  |  | Inform that one's own behavior may be an example to others |

Michie, S., Richardson, M., Johnston, M., Abraham, C., Francis, J., Hardeman, W., Eccles, M. P., Cane, J., & Wood, C. E. (2013). The behavior change technique taxonomy (v1) of 93 hierarchically clustered techniques: Building an international consensus for the reporting of behavior change interventions. *Annals of Behavioral Medicine*, *46*(1), 81–95. https://doi.org/10.1007/s12160-013-9486-6
